# Acceptability and feasibility of cognitive assessments with adults with primary brain cancer and brain metastasis: A systematic review

**DOI:** 10.1093/nop/npac097

**Published:** 2022-12-26

**Authors:** Melissa A Carlson, Elizabeth A Fradgley, Della Yates, Sarah Morris, Jordan Tait, Christine L Paul

**Affiliations:** School of Medicine and Public Health, College of Health, Medicine, and Wellbeing, University of Newcastle, Callaghan, NSW 2308, Australia; School of Medicine and Public Health, College of Health, Medicine, and Wellbeing, University of Newcastle, Callaghan, NSW 2308, Australia; School of Medicine and Public Health, College of Health, Medicine, and Wellbeing, University of Newcastle, Callaghan, NSW 2308, Australia; School of Medicine and Public Health, College of Health, Medicine, and Wellbeing, University of Newcastle, Callaghan, NSW 2308, Australia; GP Synergy, NSW & ACT Research and Evaluation Unit, Level 1, 20 McIntosh Dr, Mayfield West, NSW 2304, Australia; School of Medicine and Public Health, College of Health, Medicine, and Wellbeing, University of Newcastle, Callaghan, NSW 2308, Australia

**Keywords:** acceptability, brain cancer, cognition, cognitive assessment, easibility

## Abstract

Routine cognitive assessment for adults with brain cancers is seldom completed but vital for guiding daily living, maintaining quality of life, or supporting patients and families. This study aims to identify cognitive assessments which are pragmatic and acceptable for use in clinical settings. MEDLINE, EMBASE, PsycINFO, CINAHL, and Cochrane were searched to identify studies published in English between 1990 and 2021. Publications were independently screened by two coders and included if they: (1) were peer-reviewed; (2) reported original data relating to adult primary brain tumor or brain metastases; (3) used objective or subjective assessments; (4) reported assessment acceptability or feasibility. The Psychometric And Pragmatic Evidence Rating Scale was used. Consent, assessment commencement and completion, and study completion were extracted along with author-reported acceptability and feasibility data. PROSPERO Registration: CRD42021234794. Across 27 studies, 21 cognitive assessments had been assessed for feasibility and acceptability; 15 were objective assessments. Acceptability data were limited and heterogeneous, particularly consent (not reported in 23 studies), assessment commencement (not reported in 19 studies), and assessment completion (not reported in 21 studies). Reasons for non-completion could be grouped into patient-factors, assessment-factors, clinician-factors, and system-factors. The three cognitive assessments with the most acceptability and feasibility data reported were the MMSE, MoCA, and NIHTB-CB. Further acceptability and feasibility data are needed including consent, commencement and completion rates. Cost, length, time, and assessor burden are needed for the MMSE, MoCA, and NIHTB-CB, along with potentially new computerized assessments suited for busy clinical settings.

## Rationale

Brain cancer is life threatening and debilitative.^1^ Primary brain cancers have no lifestyle risk factors, low survival rates, and high incidence of chronic disability among survivors.^2,3^ Primary brain cancers account for 2% of all cancer diagnoses internationally. The incidence of brain metastases from other primary cancers is estimated to be 9–17%.^4,5^ Similar to primary brain cancers, brain metastases are characterized by low survival and disability.^5^

### Cognitive Impairment in Primary Brain Cancer and Metastases

Cancer-related cognitive impairment is reported in up to 75% of all cancer patients at some point during treatment, and may persist in up to 35% of patients in the years following treatment.^6,7^ Cognitive impairment is estimated to affect 80% of primary brain cancer patients and 90% of patients with brain metastases at diagnosis.^8–14^ Cognitive concerns can be exacerbated by surgical, radiation or chemotherapy treatment and can result in complications such as seizures, fatigue, or mood changes.^15–17^ People with primary brain cancer or brain metastases can experience impairment in attention, working memory, and information processing speed.^15,16^

For people with low-grade brain tumors, mild cognitive impairment can impact daily living, affecting their ability to work, to live independently and to fully engage in relationships and leisure.^18,19^ People with high-grade brain cancers or metastases can experience rapid cognitive decline which hinders decision-making about care and treatment, can necessitate intensive support from informal caregivers, and reduce physical health, emotional health and quality of life.^20–24^

### Measuring Cognitive Impairment

Routine cognitive assessment with any brain cancer patient, regardless of stage, provides opportunities for cognitive interventions and supportive care referrals.^15,19,25,26^ Accordingly, there has been interest in cognitive assessments,^13^ using either objective (neuropsychological) tests or subjective assessment of perceived impairment using self-report questionnaires or interviews.^27^ The gold-standard for cognitive assessment (not specific to cancer) is a neuropsychologist-administered neuropsychological battery defined as “two or more tests that are related by an assessment method…, for a neuropsychological interpretation”.^28^ The test evaluates memory, attention, concentration, language, information processing speed, spatial ability, and psychomotor ability.^13,29–32^

Delivering gold-standard neuropsychological testing can be costly, requiring lengthy time commitments and clinical expertise.^31^ This is prohibitive in some clinical environments and can lead to selection bias toward patients who are less fatigued or experiencing little cognitive defect.^31–33^ One alternative to intensive neuropsychological batteries is brief cognitive screens, such as the Mini-Mental State Examination (MMSE) and the Montreal Cognitive Assessment (MoCA).^33^ Although these screens are more time and cost efficient, they are not designed to be sensitive to the subtle clinical changes experienced by people with brain cancers.^9,33,34^ Self-report or report-by-proxy questionnaires or interviews can also report patients’ perceived cognitive impairment.^27,35,36^ However, studies have identified incongruence between objective and subjective cognitive assessments,^11,13,37,38^ with inconsistencies in assessment administration and the definition of cognitive impairment.^27^

Different assessment approaches have specific limitations and health services wishing to select an appropriate assessment require clear and comprehensive guidance on the various tools’ attributes and outcomes. Specifically, it is essential that implementation issues such as feasibility and acceptability—including the patient perspective—are closely examined for all assessments including those that have been tested for psychometric rigor. Assessing the acceptability of a cognitive assessment for patients and carers is particularly important for people experiencing brain cancer, given the high levels of distress and life disruption associated with their disease and treatment.^18,19,23,24^

### Acceptability and Feasibility of Cognitive Assessment

Although psychometrically sound measurement is fundamental to the choice of a cognitive assessment, essential pragmatic criteria must also be considered. These include: being considered valuable to healthcare professionals; important to patients; actionable; and incurring low burden for respondents and staff.^39^

Systematic reviews have identified psychometrically-tested cognitive assessments used in brain cancer settings^40–42^ and clinical trials.^43^ These reviews reported great variability in the assessment of cognition in brain cancer settings^40,41^ and concluded that unsuitable use or improper administration of cognitive assessments were common.^43^ However, there were no reviews exploring the pragmatic aspects of cognitive assessments, especially their acceptability and feasibility for use in busy clinical settings with this vulnerable group.

This systematic literature review aims to:

(1) Identify subjective and objective cognitive assessments for which feasibility and acceptability has been reported in relation to adult primary brain cancers and metastases, and
(2) Assess the acceptability and feasibility of these assessments using a pragmatic criteria and report consent, assessment completion, and study completion rates.

## Methods

### Protocol and Registration

This review follows the Preferred Reporting Items for Systematic Reviews and Meta-Analyses (PRISMA) (Supplementary material),^44^ and is registered with the PROSPERO International Prospective Register of Systematic Reviews (CRD42021234794).

### Search Strategy

**Figure 1:**
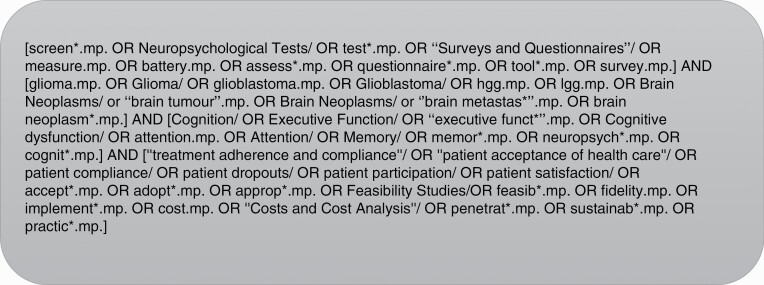
MEDLINE search strategy.

MEDLINE, EMBASE, PsycINFO, CINAHL, and Cochrane were searched for English studies published from January 1990 to December 2021. Search terms were iteratively developed using a PICO (Problem, Population, Intervention and Comparison, and Outcome) Statement.^45,46^ A senior librarian was consulted for Boolean operators, truncation, and subject headings. The final search terms related to the following key concepts: Cognition (Problem), Brain tumors and brain metastases (Population), measures and screens (Intervention and Comparison), acceptability and feasibility (Outcome). The population was refined to adult (≥18 years) brain tumors and brain populations during the screening phase. Figure 1 outlines the MEDLINE database search. The complete search strategy is in Supplementary File 1.

### Eligibility

Publications were screened by two independent coders and included if they: (1) were original peer-reviewed; (2) reported original data from a sample of adults (≥18 years in age) with a confirmed primary brain tumor or brain metastases; (3) used objective or subjective cognitive assessments; (4) reported assessment acceptability or feasibility. Discrepancies between reviewers were discussed with a third reviewer. Covidence software and Endnote X9/20 facilitated record management and screening.

### Study Selection

#### Data extraction process.

Data were independently extracted from publications by two authors (MC and EF), with any disagreements resolved through discussion. The following information was extracted:

(1) Study characteristics: author(s); title; year; country; study aims; assessment of interest; sample size; sample histology and type of brain cancer; sex or gender; age; Cultural and Linguistical Diversity; and whether the study expressly aimed to assess acceptability or feasibility. Any association between sample characteristics and assessment performance or acceptability was also recorded.
(2) Assessment(s): objective (neuropsychological) tests or subjective assessment of perceived impairment using self-report questionnaires or interviews; domains; tests/subtests; administrator; delivery (computer/paper/oral and remote); completion time.
(3) Consent, assessment, and study completion rates: Consent rates were defined as the proportion of eligible and approached individuals who consented to the study. Assessment commencement rates were defined as the proportion of eligible, consented participants who started an assessment. Assessment completion rates were defined as the proportion of individuals who started an assessment that also finished the assessment. The study completion rates were the proportion of consenting individuals who completed assessments at all study timepoints.
(4) Pragmatism according to the Psychometric And Pragmatic Evidence Rating Scale (PAPERS)^47^: The pragmatism of cognitive assessments were assessed using the pragmatic elements of PAPERS. This included: cost, language, assessor burden (ease of training), assessor burden (easy to interpret), and length (number of items).^47^ Time to complete was also extracted. If sufficient information was provided, the PAPERS’ 6-point scale (ie, poor to excellent) was used. If limited information was provided and was insufficient to score, no score was given but raw data was recorded.
(5) Patient acceptability and clinical feasibility: Acceptability was defined as self-reported or observed patient perceptions of the appropriateness of the assessment.^48^ Feasibility was defined as clinician perceptions of feasibility of assessment administration in clinical settings.^49^ Acceptability data could be quantitative or qualitative findings.^48^

#### Result synthesis.

A narrative synthesis was favored over meta-analysis given the heterogeneity of studies’ methodology and measurements.^50^ Risk of bias in individual studies was considered but not reported due to heterogeneity of research designs.

Pragmatism was synthesized according to the PAPERS components.^47^ Study and assessment completion rates were considered as indicators of both acceptability and feasibility, as the reasons for non-completion could relate to patient acceptability of the assessment or administration logistics.^48^ Patient perceptions of the appropriateness of the assessment were considered to be indicators of patient acceptability.^48^ Clinician perspectives of feasibility of assessment administration were considered to be indicators of clinical feasibility.^49^

## Results

### Study Selection

**Figure 2.**
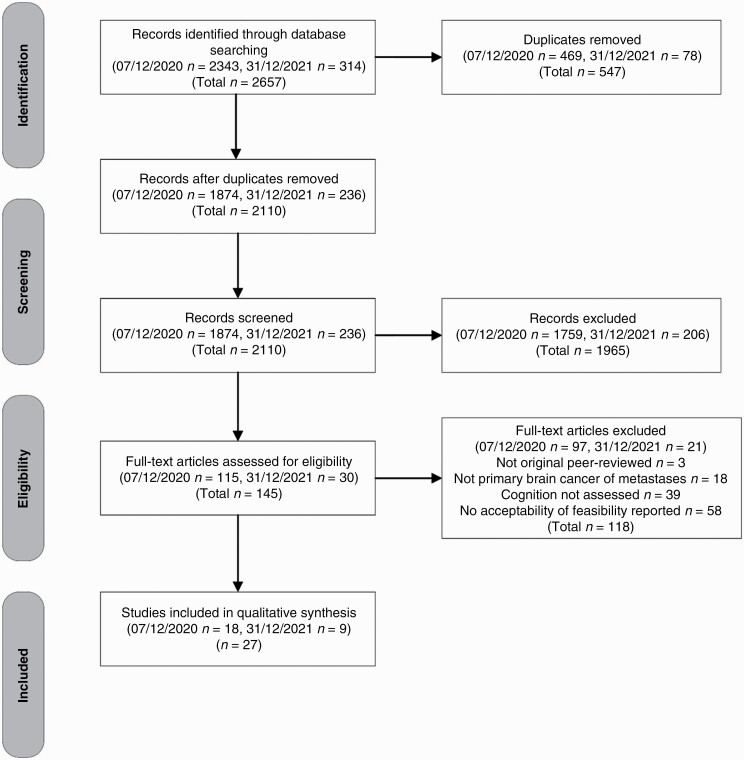
PRISMA diagram.

The initial search was conducted on 7 December 2021 (S1) with a second search conducted on 31 December 2021 (S2). Database searches yielded 2657 records (S1 *n* = 2343, S2 *n* = 314). Of these, 547 duplicate records were excluded (S1 *n* = 469, S2 *n* = 78). The remaining 2110 records were independently screened (MC, SM, and JT) with 1965 (S1 *n* = 1759, S2 *n* = 206) excluded. Of the 145 (S1 *n* = 115, S2 *n* = 30) full-text manuscripts screened, a total of 27 studies were included for data extraction (see Figure 2 for PRISMA diagram).

### Study Characteristics

#### Study characteristics.

One study was published prior to 2000,^51^ three studies between 2000 and 2009,^52–54^ 13 studies between 2010 and 2019,^8,55–66^ and ten studies were published in 2021 and 2022 alone.^67–76^ The studies were predominantly conducted in the United States (*n* = 8)^51–53,56,66,67,69,73^ and Germany (*n* = 4).^8,58,60,64^ Of the 27 studies, 15^8,51–56,60,63,64,66,67,70,73,76^ specified acceptability or feasibility in a study aim.

#### Sample characteristics.

Sample characteristics are outlined in Table 1. A range of brain cancer types were reported. Sex or gender was reported in all studies except for one^55^. The terms sex and gender were used interchangeably, and none reported the inclusion of intersex, non-binary, or gender-diverse participants.

**Table 1.** Study and sample characteristics

Assessment	First author	Country	Research Design	Sample Size (*n*)*	Histology	HistologyHy HystologyHi	Gender % Women:Men	Age	Language	Administered by	Delivery method	Remote
Addenbrooke’s Cognitive Examination-revised (ACE-R)^59^	Kerrigan 2014	United Kingdom	Cohort Study	100	Radiologically suspected intracranial tumor	Primary Brain Tumor	50:50	Categorical	–	Qualified member of the research team	–	–
Addenbrooke’s Cognitive Examination (ACE-III)^76^	Valiyaveettil 2021	India	Feasibility Study	20	Gliomas	Glioma	45:55	Md = 64	–	–	–	–
BCSE (also MoCA and MMSE)^60^	Becker 2016	Germany	Feasibility Study	58	Intracranial tumors (Meningioma, Astrocytoma, Glioblastoma multiforme, other, no histopathological report	Primary Brain Tumor	50:50	*Xd* = 58.03	–	–	–	–
Central Nervous System Vital Signs (CNS VS, Dutch Translation)^68^	Rijnen 2020	The Netherlands	Cohort Study	208	Glioblastoma	Glioblastoma	29:71	*X̅* = 58.5	Dutch	Self-administered	Computer	–
CNS Vital Signs (CNS VS, Dutch Translation)^65^	van Loenen 2018	The Netherlands	Cohort Study	125	Glioblastoma	Glioblastoma	33:67	*X̅* = 58.6	Dutch	Qualified member of the research team	Computer	No
Cogstate^66^	Cerhan 2019	United States	Feasibility Study	39	Anaplastic astrocytoma, Glioblastoma, Gliosarcoma, Oligodendroglioma	Glioma and Glioblastoma	37.5:62.5	Md = 57	English	Qualified member of the research team	Computer	No
FEDA, FEAG^58^	Cole 2013	Germany	Cohort Study	50	Brain metastases	Brain Metastases	56:44	Md = 56	German	Self-administered	Paper	Yes
Frontal Assessment Battery (FAB)^71^	Borde 2021	India	Cohort Study	50	frontal lobe lesions	Frontal lobe lesions	64:36	*X̅* = 54	–	–	–	–
Macarthur Competence Assessment Tool for Treatment (MacCAT-T)^67^	Occhiogrosso 2020	United States	Feasibility Study	11	Grade 3 or 4 Glioma	High-Grade Glioma	55:45	Unclear	English	Qualified member of the research team	Verbal	–
Milano-Bicocca Battery (MIBIB)^70^	Zarino 2020	Italy	Feasibility Study	102	High-Grade Glioma	High-Grade Glioma	34:66	Md = 59	Italian	Qualified member of the research team	–	–
MMSE^56^	Bae 2011	United States	Feasibility Study	143	Malignant astrocytomas	Low-grade glioma	35:65	Categorical	–	Qualified member of the research team	–	–
MMSE^51^	Choucair 1997	United States	Feasibility Study	126	Low-grade glioma (oligodendroglioma, astrocytoma, oligostrocytoma and other unspecified grade 1–2 glial tumors)	Malignant astrocytomas	37:63	Categorical	–	Qualified member of the research team	Paper	No
MMSE^57^	Yavas 2012	Turkey	Clinical Trial	43	Brain cancer— brain metastases, gbm, pure and mixed naplastic oligodedrogliomas, astrocytoma, lgg (8 separate trials)	primary brain tumor and brain metastases	44:56	Md = 53	–	Self-administered	–	–
MoCA^61^	Naehrig 2016	Australia	Cohort Study	53	Supratentorial located brain tumor	Brain Metastases	70:30	*X̅* = 56	English	–	–	–
MoCA^54^	Olson 2008	Canada	Feasibility Study	40	recurrant glioblastoma	Recurrant glioblastoma	45:55	Md = 60.76	English	–	–	–
MoCA^64^	Renovanz 2018	Germany	Feasibility Study	63	High-grade glioma; low-grade glioma; brain metastases (lung; breast; colorectal; melanoma; renal cell; other)	Supratentorial located brain tumor	40:60	Md = 62	German	Qualified member of the research team	–	–
MoCA, MMSE, Neuropsychological Battery^55^	Olson 2010	Canada	Feasibility Study	52	Brain Metastases	High-grade glioma; low-grade glioma; brain metastases	–	S1 *X̅* = 60.7, S2 *X̅* = 27.8	English	–	–	–
NeuroCogFX^8^	Fliessbach 2010	Germany	Feasibility Study	49	Tumors were grade I (*n* = 5, 10%), grade II (*n* = 27, 55%), grade III (*n* = 15, 31%), and grade IV (*n* = 2, 4%) gliomas	Glioblastoma	47:53	Md = 39	German	Qualified member of the research team	Computer/ oral	No
NeuroCogFX (COG)^75^	Tinchon 2021	Austria	Cohort Study	18	Glioblastoma	Primary Brain Tumor	40:60	Md = 64	–	–	Computer	–
Neuropsi attention and Memory (second edition)^74^	Loaiza 2021	Colombia	Clinical Trial	32	Malignant glioma	Malignant glioma	44:66	Md = 46	Spanish	Neuropsychologist	–	–
Neuropsychological Battery^73^	Gardner 2021	United States	Feasibility Study	119	High-grade primary brain tumor; Low- grade primary brain tumor; brain metastases; Non-CNS Ca/ NTXCa/NTX	glioblastoma or first recurrence of a locally diagnosed WHO grade II or III glioma	52:48	Md = 59	–	Neuropsychologist	Verbal/ paper via telehealth	Yes
Neuropsychological Battery^52^	Herman 2003	United States	Feasibility Study	30	Brain Metasteses: Lung (14); breast (7); Melanoma (4); others (5)	Primary brain tumor	57:43	Md = 56	English	Qualified member of the research team	Paper/ physical task	No
Neuropsychological Battery & Medical Outcome Study (MOS)^72^	Caramanna 2021	The Netherlands	Clinical trial	546	glioblastoma or first recurrence of a locally diagnosed WHO grade II or III glioma	primary brain tumor and brain metastases	37:63	*X̅* = 55.25	–	–	–	–
Neuropsychological Battery (included MMSE)^53^	Regine 2004	United States	Feasibility Study	370	Primary brain tumor	Brain metastases	45:55	Categorical	–	Qualified member of the research team	–	–
Neuropsychological battery focused on language domains^69^	Tibbs 2020	United States	Clinical trial	59	Primary brain tumors and brain metastases	Brain tumor and brain metastases	43:57	Md = 47	English	Neuropsychologist	–	–
NIH Health Toolbox Cognitive Battery (NIHTB-CB)^63^	Lang 2017	Canada	Feasibility Study	18	Diffuse gliomas (astrocytoma, gbm, oligodendroglioma, pleeomorphic xanthoastrocytoma	Glioma	28:72	Md = 39.5	–	Qualified member of the research team	Computer	–
Wechsler Adult Intelligence Scale- Third Edition (WAIS-II) ^62^	Gonçalves 2017	Portugal	Cohort Study	37	Brain tumors: glioblastoma, astrocytoma, oligodendroglioma, lymphoma, oligoastrocytoma, meningioma, and glioma	Primary brain tumor	36:64	*X̅* = 54.90	Portuguese	Neuropsychologist	–	No

*Reported as number consented, if consented not provided, reported as number who commenced measure, if not reported, then reported as number who completed measure.

#### Identified assessments.

A total of 21 different cognitive assessments were reported across the 27 studies. Fifteen of the 16 named assessments were objective (*n* = 15) rather than subjective (*n* = 1) assessments. The most commonly used assessments were brief cognitive screens (<15 min to complete) such as Mini-Mental State Examination (MMSE) (*n* = 5)^51,55–57,60^ and the Montreal Cognitive Assessment (MoCA) (*n* = 5).^54,55,60,61,64^ Other cognitive assessments included the Addenbrooke’s Cognitive Examination (ACE-R, and ACE-III)^59,76^ (*n* = 2), Central Nervous System Vital Signs (CNS VS)^65,68^ (*n* = 2), and NeuroCogFx (*n* = 2).^8,75^ Five studies used study-specific neuropsychological battery of tests,^52,53,69,72,73^ two of which also included either the MOS^72^ and MMSE^53^.

### *Pragmatic Characteristics of Assessments Using PAPERS* ^47^

#### Length in time (reported by nine studies).

The briefest were the Brief Cognitive State Examination (BCSE) with a median completion time of 8 min (range, 4–15 min)^60^ and the Frontal Assessment Battery (FAB) with a mean completion time of 8 min (range, 6–12 min). This was followed by the MoCA with three studies reporting the majority completed in under 10–15 min.^54,64^ The two longest assessments were the National Institutes of Health Toolbox—Cognitive Battery (NIHT-CB)^63^ and Direct To Home—Teleneuropsychology (DTH-TNP),^73^ taking over an hour.

#### Length by number of items (reported by five studies).

The number of items reported were: MMSE with 11 items,^51,56^ the MOS with six items,^72^ and the *Fragebogen erlebter Defizite der Aufmerksamkeit (*FEDA) and *Fragebogen zur Erfassung alltäglicher Ged*ä*chtnisleistungen* (FEAG) with a combined 56 items.^58^

#### Assessor burden (reported by six studies).

The training required for administering the assessment was reported for the MoCA,^64^ the NeuroCogFX,^8^ Neuropsy Attention and Memory,^74^ NIHTB-CB,^63^ and two of the neuropsychological batteries,^52,53^ one of which included the MMSE.^53^

#### Report scoring (reported by twelve studies).

Scoring was reported for the MMSE,^51,56,57^ Cogstate,^66^ the FAB,^71^ FEDA and FEAG,^58^ the CNS VS,^65^ the MoCA,^64^ Neuropsi attention and Memory,^74^ NIHTB-CB,^63^ and the Wechsler Adult Intelligence Scale-Third Edition (WAIS-II),^62^ as well as one neuropsychological battery.^52^

#### Cut-off for cognitive decline (reported by twelve studies).

A cut-off for cognitive decline was reported for the ACE-III,^76^ BCSE,^60^ FEDA,^58^ MIBIB,^70^ MMSE,^56^ MoCA,^54,55,61,64^ NeuroCogFX,^8^ the Neuropsi Attention and Memory (second edition),^74^ and the NIHTB-CB.^63^

#### Cost (reported by two studies).

Cost was reported for the MoCA^54^ and the NIHTB-CB^63^, both of which were reported to be freely available for use.

#### Readability (six studies).

No studies reported the readability of the assessment. The Flesch-Kincaid score was obtained and assessed in accordance to the PAPERS readability criteria for all assessments in the public domain excluding neuropsychological batteries due to their complexity.^47^ Six assessments were able to be assessed using a Flesch-Kincaid score. Of those, the ACE-R,^59^ ACE-III,^76^ FEDA and FEAG,^58^ MMSE,^51,56,57^ and MoCA^54,61,64^ had excellent readability (≤7.9), and the FAB^71^ had good readability (8.0–12.99).

#### Sample size, consent, recruitment, and completion rates.

Sample size, consent rates, recruitment rates, assessment completion rates, and study completion rates are reported in Table 3.

**Table 2.** Pragmatic characteristics using PAPERS^47^

Assessment	Author	Cost	PAPERS cost	PAPERS language	PAPERS language	Reports training? (assessor burden)	PAPERS assessor burden ease of training	Length (# items)	PAPERS length	Report scoring?	Cut-Off for cognitive decline reported?	PAPERS assessor burden easy to interpret
Addenbrooke’s Cognitive Examination- revised (ACE-R)^59^	Kerrigan	–*	None*	4.6	Excellent	–	None	–	–	–	–	None
Addenbrooke’s Cogntiive Examination (ACE-III)^76^	Valiyaveettil	–	None	4.3	Excellent	–	None	19	Good	Yes	Yes	Good
BCSE (also MoCA and MMSE)^60^	Becker	–	None	–	None	–	None	–	-	-	Yes	Good
Central Nervous System Vital Signs (CNS VS, Dutch Translation)^68^	Rijnen	–	None	–	None	–	None	–	–	–	–	None
CNS Vital Signs (CNS VS)^65^	van Loenen	–	None	–	None	–	None	–	–	Yes	–	Good
Cogstate^66^	Cerhan	–	None	–	None	–	None	–	–	Yes	–	Minimal/emerging
FEDA, FEAG^58^	Cole	–	None	7.6	Excellent^4^	–	None	56 (FEDA1 = 13; FEDA 2 = 8; FEDA3 = 6; FEAG = 29)	Adequate	Yes	FEDA = Yes, FEAG = No	FEDA = Good FEAG = Adequate
FAB^71^	Borde	–	None	8.6	Good	–	None	–	–	Yes	No	Good
MacCAT-T^67^	Occhiogrosso	–	None	–	None	–	None	–	–	–	No	Minimal/ emerging
MIBIB^70^	Zarino	–	None	–	None	–	None	–	–	–	Yes	Minimal/ emerging
MMSE^56^	Bae	–	None	5.3	Excellent	–	None	11	Good	Yes	Yes	Good
MMSE^51^	Choucair	–	None	5.3	Excellent	–	None	11	Good	Yes	–	None
MMSE^57^	Yavas	–	None	5.3	Excellent	–	None	–	–	Yes	–	Minimal/emerging
MoCA^61^	Naehrig	–	None	0.7	Excellent	–	None	–	–	–	Yes	Good
MoCA^54^	Olson (2008)	Yes (free)	Excellent	0.7	Excellent	–	None	–	–	–	Yes	Good
MoCA^64^	Renovanz	-	None	0.7	Excellent	Yes	Good			Yes	Yes	Good
MoCA, MMSE, Neuropsychological Battery^55^	Olson (2010)	Yes (MoCA)	None	–	n/a	No	None	–	–	–	–	None
NeuroCogFX^8^	Fliessbach	–	None	–	None	Yes	Poor	–	–	–	Yes	Good
NeuroCogFX (COG)^75^	Tinchon	–	None	–	None	–	None	–	–	–	–	None
Neuropsi attention and Memory (second edition)^74^	Loaiza	–	None	–	None	Yes	Poor	–	–	Yes	Yes	Good
Neuropsychological Battery^73^	Gardner	–	None	–	n/a	–	None	–	–	–	–	None
Neuropsychological Battery^52^	Herman	–	None	–	n/a	Yes	Adequate	–	–	Yes	–	None
Neuropsychological Battery & Medical Outcome Study (MOS)^72^	Caramanna	-	None	-	n/a	-	None	6 (MOS)	Good (MOS)	–	–	None
Neuropsychological Battery (included MMSE)^53^	Regine	–	None	–	n/a	Yes	Adequate	–	–	–	–	None
Neuropsychological battery focused on language domains^69^	Tibbs	–	None	–	n/a	–	None	–	–	–	–	None
NIH Health Toolbox Cognitive Battery (NIHTB-CB)^63^	Lang	Yes	Excellent	–	None	Yes	Adequate	–	Adequate	Yes	Yes	Good
Wechsler Adult Intelligence Scale- Third Edition (WAIS-II) ^62^	Gonçalves	–	None	–	None	–	None	–	–	Yes	–	Adequate

*‘–’ Indicates the criterium was not reported in the table. “None” indicates the study was assessed and scored as “none” using the PAPERS criteria. Based on a translated excerpt due to copyright .

**Table 3.** Consent, commencement, assessment completion, and study completion rates

Assessment	First author	Sample Size (*n*)^1^	Approached (*n*)	Consented (*n*)	Consent rate^2^	Number of times administered	Started assessment (*n*)	Commencement rate^3^	Finished assessment (*n*)	Assessment completion rate^4^	Completed all timepoints (*n*)	Study completion rate^5^
ACE-R^59^	Kerrigan	100	–	100	–	1	–	–	100	–	n/a	–
ACE-III^76^	Valiyaveettil	20	–	–	–	1	–	–	20	–	n/a	–
BCSE. MoCA, MMSE^60^	Becker	58	–	–	–	1	–	–	58	–	n/a	–
CNS VS, Dutch ^68^	Rijnen	208	–	–	–	2	–	–	208	–	136	65%
CNS VS^65^	van Loenen	125	147	–	–	2	–	–	125	–	82	66%
Cogstate^66^	Cerhan	39	–	–	–	2	T1: 39, T2: 27	–	T1: 21, T2: 23	–	27	–
FEDA, FEAG^58^	Cole	50	68	–	–	4	–	–	50	–	22	44%
FAB^71^	Borde	50	–	–	–	1	–	–	50	–	n/a	–
MacCAT-T^67^	Occhiogrosso	11	–	–	–	1	–	–	11	–	n/a	–
MIBIB^70^	Zarino	102	–	102	–	5	102	100%	80	78%	18/26	–
MMSE^56^	Bae	143	–	–	–	Varies within studies	–	–	–	–	143	–
MMSE^51^	Choucair	126	–	126	–	–	119	94%	119	100%	–	–
MMSE^57^	Yavas	43	–	–	–	4	–	–	43	–	21	49%
MoCA^61^	Naehrig	53	–	53	–	1	–	–	50	–	n/a	–
MoCA^54^	Olson (2008)	40	–	40	>90%	1	40	100%	40	100%	n/a	–
MoCA^64^	Renovanz	63	–	63	–	2	63	100%	63	100%	63	100%
MoCA, MMSE, Neuropsychological Battery^55^	Olson (2010)	52	92	52	57%	1	1?	–	36	–	n/a	–
NeuroCogFX^8^	Fliessbach	49	–	–	–	2	49	–	45	92%	–	–
NeuroCogFX ^75^	Tinchon	18	–	–	–	1	–	–	18	–	n/a	–
Neuropsi attention and Memory (second edition)^74^	Loaiza	32	–		–	2	–	–	32	–	16	50%
Neuropsychological Battery^73^	Gardner	119	–	119	–	4	79	66%	–	–	–	–
Neuropsychological Battery^52^	Herman	30	30	30	100%	4	30	100%	Unclear: 9 unable to completed pegboard, 2 unable to complete trailmaking A, 8 unable to complete trailmaking B	–	T2=10, T3 = 4, T 4 = 2, no refusals	–
Neuropsychological Battery and Medical Outcome Study (MOS)^72^	Caramanna	546	–	546	–	1	546	100%	–	–	n/a	–
Neuropsychological Battery,MMSE^53^	Regine	370	–		–	3	–	–	370	–	T1: 155/261 alive pts; T2 83/213 alive pts	–
Neuropsychological battery focused on language domains^69^	Tibbs	59	–	59	–	4	–	–	Unclear	–	–	–
NIHTB-CB^63^	Lang	18	–	18	–	2	18	100%	18	100%	13	72%
WAIS-II ^62^	Gonçalves	37	76	37	49%	1	–	–	23	–	n/a	–

^1^Reported as number consented, if consented not provided, reported as number who commenced measure, if not reported, then reported as number who completed measure.

^2^As provided in manuscript, if not provided, calculated as the number of people consented divided by the number of people who were approached to participate.

^3^As provided in manuscript, if not provided, calculated as the number of people who started an assessment, divided by the number of people who consented to the study.

^4^The number of people who completed an assessment, divided by the number of people who started the assessment.

^5^The number of people who completed all timepoints, divided by the number of people who finished one assessment.

#### Sample size.

Sample sizes ranged from 11 to 546 participants, with a mean of 95 participants and a median of 52 participants.

#### Consent rates (reported by four studies).

The highest consent rates were for an unnamed neuropsychological battery (100%)^52^ and the MoCA alone (>90%).^54^ One study reported reasons for declining consent, which was primarily not wanting to participate in a four hour neuropsychological assessment.^55^

#### Commencement rates (reported by eight studies).

The highest proportion of consented participants starting the assessment were the MoCA (100%),^54,64^ the MIBIB (100%),^70^ and the NIHTB-CB (100%),^63^ an unnamed neuropsychological battery (100%)^52^ and neuropsychological battery plus MOS (100%).^72^ These were followed by the MMSE (94%),^51^ and an unnamed neuropsychological battery (66%).^73^

Reasons for not starting the assessment included: patient decline or not providing informed consent^55,59,61^; patient too impaired or unwell^51,55,61,73^; institutional error or logistic problems^56,59,61^; canceled appointments^51,65^; lost to follow up after scheduling assessment administration^62^; remote administration was not appropriate for the patient or patient lacked necessary equipment or time^73^; clinician not confident the referral question could be answered using tests^73^; testing was too burdensome^65^; or participant failed to follow instructions.^66^

#### Assessment completion rates (reported by six studies).

The highest completion rates were for the MoCA (100%),^54,64^ the MMSE (100%),^51^ and the NIHTB-CB (100%),^63^ followed by the NeuroCogFX (92%),^8^ and the MIBIB (78%).^70^

Reasons for not completing the NeuroCogFX included comprehension difficulties and technical difficulties due to pressing keys too long.^8^ Non-completion of the MIBIB was considered to be due to significant cognitive impairment.^70^ Reasons for non-completion of an unnamed neuropsychological battery included patient frustration, physical disabilities or timing-out.^52^

### Study Completion Rates (Reported by Seven Studies)

Assessments were administered at more than one timepoint in 14 studies: seven studies at two timepoints,^8,63–66,68,74^ one study at three timepoints,^53^ four studies at four timepoints,^52,57,58,69,73^ one study at five timepoints,^70^ and one study administered the assessment at eight timepoints.^57^ The assessment with the highest study completion rate was the MoCA (100% completed at two timepoints).^64^

Study attrition reasons reported included: deterioration in health^52,53,56,58,65,66,68^; death^52,58,65,66,68^; institutional error^53,56^; study materials not returned^58^; testing was too burdensome^65^; not seen again at study centre^53,65,66^ or canceled appointment,^65^ and change in eligibility.^68^

#### Patient-reported acceptability.

##### BCSE Relative to MMSE and MOCA (Reported by One Study).

Patient acceptability of the BCSE was assessed using eleven-point tolerability and strain visual analogue scales ranging from 0 (not at all tolerable, not at all exhausting) to 10 (very tolerable, very exhausting).^60^ Participants (*n* = 58) were also asked which assessment (BCSE, MMSE, and MoCA) was preferred and accurately captured changes in cognition. The BCSE, MMSE, and MoCA were all reported to be equally appropriate. Relative to the MMSE and MoCA, the BCSE was preferred by 17% of patients.^60^

##### MMSE and MoCA.

One study^54^ used a five-point scale to rate the inconvenience of the MoCA and the MMSE. In the sample of 40 patients, 37 (93%) stated the MoCA was either not at all or only mildly inconvenient. Patients gave similar average inconvenience scores of both the MoCA and the MMSE.^54^

##### MoCA.

One study^64^ asked patients to complete structured interviews assessing acceptability of the MoCA at two timepoints. The MoCA was generally well accepted with most patients responding that they did not find the assessment: too burdensome (t1 90%, t2 91%); too extensive (t1 93%, t2 86%); or difficult to understand (t1 95%, t2 86%). A large proportion also reported they understood the meaning of the test (t1 89%, t2 91%), and the test was useful (t1 91%, t2 98%). However, some patients reported feeling distracted during assessments (t1 39%, t2 32%).^64^

##### Cogstate (One Study).

Patient acceptability of Cogstate was assessed using a likeability scale of 0 to 10, with the higher score being more enjoyable.^66^ Mean likeability for Cogstate was 6.9 (SD = 2.0) at the first timepoint, and 6.54 (SD = 1.8) at the second timepoint. There was no significant difference in likeability between Cogstate and a pen and paper battery; with an equal number preferring each mode of administration.

##### MacCAT-T (One Study).

Patient acceptability of the MacCAT-T was assessed by asking participants (*n =* 10) their level of distress while completing the assessment on a 10-point scale, and whether they increased their knowledge about treatment on a five-point Likert scale.^67^ The mean distress score was three (range, 1–8), and nine participants either agreed or strongly agreed that their knowledge of treatment increased after taking the MacCAT-T.^67^

##### DTH-TNP (One Study).

Patient acceptability was assessed using five-point Likert scales. In the sample of 52 respondents: 98% indicated satisfaction with the virtual assessment; 92% would recommend the virtual assessment to others; 100% of respondents felt understood by the examiner; 90% reported no technical difficulties; 94% reported no communication challenges; and 94% reported no privacy concerns.^73^

Additionally, participants reported a range of benefits for in-person and virtual assessment. For in-person assessment, benefits included: improved personal connection (40%); improved communication with examiner (17%); more extensive assessment (23%); and easier to express concerns (8%). For virtual assessment, benefits included: reduced travel time (88%); reduced risk of infection (79%); reduced anxiety (27%); and improved concentration without an examiner present (23%).

#### Clinician feasibility.

##### DTH-TNP (One Study).

Clinicians reported achieving the intended goal of the assessment in 88% of clinical encounters and partially achieving goals in 10% of evaluations.^73^ Challenges reported while administering DTH-TNP included patient dysregulation (16%), slow or unreliable internet (15%), problems with technology (9%), and test interruptions (10%). When asked about strategies used to optimize the DTH-TNP or overcome specific challenges, strategies were reported as unnecessary (54% of evaluations), or assistance from another person or family member (21%) or taking frequent breaks (9%).^73^

##### MoCA (One Study).

Feasibility for the MoCA was assessed using structured observations which included: time needed for assessment; severity of observed difficulties; and kind of difficulties observed.^64^ Moderate or major difficulties were observed in 27% of participants at timepoint 1 and 41% of participants at timepoint 2. These included physical problems (t1 25%, t2 44%), communication (t1 32%, t2 37%), assessment too extensive (t1 5%, t2 11%), complexity (t1 17%, t2 29%), instructions (t1 51%, t2 41%), and external disruptions (t1 11%, t2 10%).^64^

#### Suitability with across patient groups/bias (four studies).

Four studies reported suitability across patient groups/bias. These included demographic and clinical characteristics. No studies reported acceptability or suitability related to Culturally and Linguistically Diverse Groups.

##### CNS Vital Signs.

This study found no significant differences between the demographic and clinical characteristics of those who completed both assessments (pre- and post-operative) and those who dropped out of the study.^65^

##### Cogstate.

One study found no associations between age, gender, and likeability between the Cogstate and the paper and pen battery.^66^

##### MMSE.

This study hypothesized that clinical and demographic characteristics may be associated with patterns of missing data.^56^ However, the authors did not find an association between baseline scores and patterns of missing data.^56^

##### MMSE, NPA, and MoCA (Olson 2010).

This study reported any selection bias in who was invited and who consented to the neuropsychological assessment.^55^ The study found significant differences in age, education, dexamethasone use, MMSE and MoCA scores (*P* < .001). Furthermore, those completing neuropsychological assessments were associated with higher cognitive scores and educational status, as well as lower age, dexamethasone use, and opioid use. Individuals who completed the neuropsychological assessment had higher MoCA scores than individuals who were not asked to complete the NPA (24.7 vs. 20.5; *P* < .001).

#### Synthesized acceptability, feasibility and pragmatism across identified assessments.

Overall, the three assessments with the most comprehensive data about acceptability and feasibility were the MoCA, the NIHTB-CB, and the MMSE. Table 4 provides a summary of the acceptability of these three assessments. Those with the least acceptability and feasibility data reported were the ACE-R,^59^ ACE-III,^76^ and the FAB.^71^

**Table 4.** Summary of acceptability for assessments with most data: MoCA, NIHTB-CB, and MMSE

	MMSE	MoCA	NIHTB-CB
Number of studies	3^51,56,57^	3^54,61,64^	1^63^
Language	–	English,^54,61^ German^64^	–
Sample size	143,^56^ 126,^51^ and 43^57^	63^64^, 53^61^, and 40^54^	18^63^
Consent, commencement, assessment completion and study completion rates	Commencement rate: 94% ^51^ Assessment completion: 100% ^51^ Study completion: 49% at four timepoints^57^	Consent: >90%^54^, Recruitment: 100%^54,64^ Assessment Completion: 100%)^54,64^ Study completion: 100% at 2 timepoints^64^	Commencement and assessment completion rates: 100%^63^ Study completion: 72% at 2 timepoints^63^
Length (time)	–	98% completion in less than 15 minutes (98%)^54^ Median completion in 11 min (range, 6–26 min)^64^	35 min^63^
Length (items)	11 items^51,56^ and characterized as good using the PAPERS scale	–	–
Training (assessor burden)	–	Unclear	Online training, practice under supervision of neuropsychologist^63^
Cost	–	–	Reported as free^63^
Scoring	Scoring was reported for the MMSE^51,56,57^	–	–
Acceptability and feasibility	Patterns of missing associated with age, disease prognosis, education level, and aggressiveness of therapy. There was no association between baseline scores and patterns of missing data^56^	Not at all/ mildly inconvenient, not burdening, not too extensive. Patients understood the task, understood the meaning of the test, and found the test useful^54,64^ No/minor difficulties in 73% of administrations, moderate difficulties in 14%, and major difficulties in 13% Post-operatively major difficulties in 22% of administrations, particularly with complexity and extent of the assessment^64^	–

## Discussion

### Acceptable and Feasible Assessments

This review of 27 studies identified 21 cognitive functional assessments for which feasibility and acceptability specific to adults with primary brain cancers and brain metastases had been reported. Of note, only one assessment focused on subjective cognitive assessment. Self-reported assessment of cognition may have poor precision; however, understanding patients’ self-perceived level of competency is relevant to understanding potential desire for supportive care and has foundations in patient-centered approaches to care.^77^

Although none of the studies provided all components of the PAPERS scale nor completion data, the three assessments with the most acceptability data available were the MMSE, the MoCA, and the NIHTB-CB. These assessments are also known to have reasonable reliability and validity.^33,78,79^ For health services seeking to implement a cognitive assessment within routine practice, each of the three assessments have key attributes which may be advantageous. For those health services seeking a short test and do not have access to a neuropsychiatrist, the MMSE and MoCA may be of interest. The MMSE has 11 items,^51^ does not need to be administered by a neuropsychiatrist,^51,56,57^ achieves high levels of commencement and completion.^51^ However, repeat administrations may be challenging.^57^ Of the three domains most impacted by brain cancer (attention/executive function, processing speed, and working memory), the MMSE measures attention only.^51,56,57^ Although the MMSE had sufficient acceptability data available, it is now protected by copyright, which may limit its clinical utility.^80,81^

The MoCA is similarly brief, taking less than 15 min to complete,^61,64^ and has high levels of commencement and completion, including completion at two timepoints. The MoCA also has a cut-off for cognitive decline,^64^ is reported as free,^54^ and has good acceptability and feasibility.^54,64^ Of the three domains most impacted by brain cancer (attention/executive function, processing speed, and working memory), the MoCA measures attention only.^54,64^

For those health services capable of implementing a longer test, the NIHTB-CB is moderate in length and requires 35 min to be administered using a computer.^63^ All of the participants who started the assessment were able to complete it, and 72% of participants were able to complete it at two timepoints.^63^ A trained non-neuropsychiatrist was able to administer the assessment.^63^ Although the domains for the NIHT-CB were not reported in the included study,^63^ the NIHTB-CB measures all three domains most commonly impacted by brain cancers, being attention/executive function, processing speed, and working memory.^78^

### Psychometric Properties of the MMSE, MoCA and NIHTB-CB

It was beyond the scope of this review to assess psychometric properties (see^40–43^ for validated assessments). However, it is worth noting that the three assessments with the most acceptability data, do not have robust psychometric data specific to people with brain cancer. The MMSE and MoCA may lack the sensitivity required to detect subtle clinical changes experienced by people with brain cancers,^9,33,34^ and the NIHTB-CB has not been validated with people with brain cancers. Therefore, although these three tools have the most acceptability data available, the MMSE and MoCA may not be suitable for detecting subtle impairment in this patient group, and further research is needed to understand if the NIHTB-CB is suitable for detecting cognitive impairment (either subtle or more severe) in this patient group.

### Options Exist for Remote Administration

Remote completion of patient-reported outcome measures can be facilitated by computerized assessments. Remote assessment has the potential to enhance equity of access and move assessment away from time-poor and stressful clinical settings.

Computerized assessments were considered acceptable and feasible.^8,63,65,66,68,75^ In particular, the computerized CNS Vital Signs was completed irrespective of age, education, sex, tumor location, tumor size, use of antiepileptic and corticosteroid drugs, and treatment.^65^ Cogstate was reported as acceptable by patients and an equal number of participants preferred the computerized Cogstate to a paper assessment; this was irrespective of age or gender.^65^ Similarly, the DTH-TNP—administered virtually—was considered to be acceptable by participants and feasible by clinicians.^73^

### Improvement Opportunities

#### Limited reporting of acceptability data and consent rates.

—One key finding of this review is the limited number of studies that noted patient-reported acceptability (seven of 27 studies) or clinician feasibility (two of 27 studies). It is critical that assessment approaches are considered valuable by clinicians and do not confer excessive burden for patients.^39^ Studies that describe the patient experience of completing cognitive assessments, alongside the clinical utility of the results, are needed.

Reporting of consent, commencement or completion rates was not consistent across studies. This presented a challenge for comparing assessments and for understanding the reasons for non-completion.^38,56^ Where reported, common themes affecting non-completion emerged. These themes could be grouped into patient-factors (eg, patient deterioration), assessment-factors (eg, unclear instructions or testing was perceived to be too burdensome), clinician-factors (eg, unsure of result utility), and system-factors (eg, institutional error). Assessment-factors were common across the tools suggesting the content of cognitive assessment require careful consideration. Furthermore, large-scale adoption of a cognitive assessment will require implementation strategies targeted at each level, such as patient coaching to complete assessment, clinician education to emphasize benefit, and institutional support or funding.

#### Equitable inclusion and reporting of minority groups in clinical research.

—In the 27 studies, there was limited reporting of cultural and linguistic diversity. Sex or gender was reported in all but one study with equitable representation of women in most (*n* = 17) studies, however, no studies reported the inclusion of intersex or non-binary participants. Racial and ethnic minorities, Indigenous peoples, culturally and linguistically diverse people, and people who are not men have all been historically underrepresented in health research.^82–85^ Given brain cancer indiscriminately affects people irrespective of gender, age, and cultural and linguistic diversity, this lack of inclusion can result in inaccurate generalizing of non-inclusive data to these populations.^85,86^

### Limitations

This review did not report on psychometric characteristics of the assessments as this information can be found in other systematic reviews.^40–43^ This review focused specifically on cognitive assessment in brain cancer to the exclusion of assessments in use for other tumor types. Only 15 of papers in this review expressly aimed to assess acceptability and feasibility, requiring some interpretation for the other papers, and resulting in data which were heterogeneous, unclearly defined, and missing in many areas of interest.

## Conclusion

Several cognitive assessments have been reported as acceptable and feasible for use with adults with primary brain cancers and brain metastases, with the most comprehensively reported being the MMSE, MoCA, and NIHTB-CB. However, the NIHTB-CB has not yet been validated with brain cancers, and the MMSE and MoCA are not sensitive enough to detect subtle changes in cognition in this population. Therefore, this study makes no specific recommendations for a tool for clinical use. However, further acceptability and feasibility data with adults with brain cancer such as consent, assessment commencement, assessment completion, study completion rates, and reasons for study decline, assessment incompletion, or study withdrawal, language, cost, length, time to administer, and assessor burden are needed for the MMSE, MoCA, and NIHTB-CB, along with potentially new assessments suited for busy clinical settings. Further studies are needed to identify whether other known assessments are acceptable and feasible as cognitive assessments for people with brain cancer and fit for routine clinical use to facilitate assistance with daily living and quality of life for patients and families.

## Supplementary Material

npac097_suppl_Supplementary_Material
