# Supplementary material for: Acceptability and feasibility of cognitive assessments with adults with primary brain cancer and brain metastasis: A systematic review
Source: Neurooncol Pract. 2022 Dec 26;10(3):219–37. doi: 10.1093/nop/npac097 (PMC10180383; doi:10.1093/nop/npac097)
Supplement: npac097_suppl_Supplementary_Material [file npac097_suppl_supplementary_material.docx]

# Supplementary File 1

## MEDLINE

[screen*.mp. OR Neuropsychological Tests/ OR test*.mp. OR “Surveys and Questionnaires”/ OR measure.mp. OR battery.mp. OR assess*.mp. OR questionnaire*.mp. OR tool*.mp. OR survey.mp.] AND [glioma.mp. OR Glioma/ OR glioblastoma.mp. OR Glioblastoma/ OR hgg.mp. OR lgg.mp. OR Brain Neoplasms/ or “brain tumour”.mp. OR Brain Neoplasms/ or “brain metastas*”.mp. OR brain neoplasm*.mp.] AND [Cognition/ OR Executive Function/ OR “executive funct*”.mp. OR Cognitive Dysfunction/ OR attention.mp. OR Attention/ OR Memory/ OR memor*.mp. ORneuropsych*.mp. OR cognit*.mp.] AND ["treatment adherence and compliance"/ OR "patient acceptance of health care"/ OR patient compliance/ OR patient dropouts/ OR patient participation/ OR patient satisfaction/ OR accept*.mp. OR adopt*.mp. OR approp*.mp. OR Feasibility Studies/ OR feasib*.mp. OR fidelity.mp. OR implement*.mp. OR cost.mp. OR "Costs and Cost Analysis"/ OR penetrat*.mp. OR sustainab*.mp. OR practic*.mp.]

## EMBASE

[screen.mp. OR neuropsychological test/ OR test.mp. OR survey.mp. OR questionnaire/ OR questionnaire.mp. OR measure.mp. OR battery.mp. OR clinical assessment tool/ OR tool.mp. OR cognition assessment/ OR cognition/] AND glioma/ or glioma.mp. OR glioblastoma.mp. OR glioblastoma/ OR hgg.mp. OR lgg.mp. OR brain tumour.mp. or brain tumor/ OR brain metastasis/ OR brain metastas*.mp. OR brain neoplasm.mp.] AND [cognition/ or cognit*.mp. OR cognition.mp. OR cognition/ OR numerical cognition/ OR social cognition/ OR executive function/ OR executive funct*.mp. OR attention/ OR attention.mp. OR memory/ OR memor*.mp. OR neuropsychiatry/ OR neuropsychological test/ OR cognitive defect/ OR neuropsych*.mp. OR neuropsychology/] AND [treatment adherence.mp. OR patient compliance/ OR patient satisfaction.mp. OR patient satisfaction/ OR accept*.mp. OR adopt*.mp. OR approp*.mp. OR feasibility study/ or feasib*.mp. OR fidelity.mp. OR implement*.mp. OR "cost benefit analysis"/ OR "cost"/ OR "cost effectiveness analysis"/ OR "program cost effectiveness"/ OR cost.mp. OR "cost utility analysis"/ OR penetrat*.mp. OR sustainab*.mp. OR practic*.mp.]

## PsycINFO

[exp Screening/ or screen*.mp. OR test*.mp. OR measure*.mp. OR exp Surveys/ or survey*.mp. OR exp Test Battery/ OR battery*.mp. OR assess*.mp. OR exp Questionnaires/ OR questionnaire*.mp. OR exp Measurement/ OR tool*.mp.] AND [glioma.mp. OR exp Glioma/ OR glioblastoma.mp. OR hgg.mp. OR lgg.mp. OR exp Brain Neoplasms/ or "brain tumour".mp. OR brain metastases.mp. OR brain neoplasm*.mp.] AND [exp Cognitive Ability/ OR exp Cognition/ OR cognit*.mp. OR executive OR funct*.mp. OR exp Attention/ OR attention.mp. OR exp Memory/ or memor*.mp. OR exp Neurocognition/ OR exp Neuropsychology/ OR neuropsych*.mp.] AND [exp Client Attitudes/ OR accept*.mp. OR adopt*.mp. OR approp*.mp. OR feasibility.mp. OR fidelity.mp. OR implement*.mp. OR exp "Costs and Cost Analysis"/ OR cost*.mp. OR penetration.mp. OR sustainable.mp. OR sustainability.mp. OR practic*.mp.]

## CINAHL

[exp Screening/ OR screen*.mp. OR test*.mp. OR measure*.mp. OR exp Surveys/ OR survey*.mp. OR

exp Test Battery/ OR battery*.mp.] AND [glioma.mp. OR exp Glioma/ OR glioblastoma.mp. OR hgg.mp. OR lgg.mp. OR exp Brain Neoplasms/ OR "brain tumour".mp. OR brain metastases.mp. OR brain neoplasm*.mp. OR assess*.mp. OR exp Questionnaires/ OR questionnaire*.mp. OR exp Measurement/ OR tool*.mp.] AND [exp Cognitive Ability/ OR exp Cognition/ OR cognit*.mp. OR executive funct*.mp. OR exp Attention/ OR attention.mp. OR exp Memory/ OR memor*.mp. OR exp Neurocognition/ OR exp Neuropsychology/ OR neuropsych*.mp.] AND [exp Client Attitudes/ OR accept*.mp. OR adopt*.mp. OR approp*.mp. OR feasibility.mp. OR fidelity.mp. OR implement*.mp. OR exp "Costs and Cost Analysis"/ OR cost*.mp. OR penetration.mp. OR sustainable.mp. OR sustainability.mp. OR practic*.mp.]

## Cochrane

[test* OR screen* OR Weights and Measures/ OR measure* OR battery* OR Luria-Nebraska Neuropsychological Battery/ OR Independent Medical Evaluation/ OR assess* OR Surveys and Questionnaires/ OR Questionnaire* OR tool*] AND [Glioma/ OR glioma OR Glioblastoma/ OR glioblastoma OR hgg OR lgg OR brain tumour OR Brain Neoplasms/ OR brain neoplasms] AND [Executive Function/ OR executive funct* OR Attention/ OR memor* OR Memory Disorders/ neuropsych* OR cognit* OR Cognition/] AND [accept* OR adopt* OR Program Evaluation/ OR approp* OR feasib* OR Feasibility Studies/ OR fidelity OR implement* OR Costs and Cost Analysis/ OR cost* OR penetrat* OR sustainab* OR practic*]
